# The influence of pH and divalent/monovalent cations on the internal electron transfer (IET), enzymatic activity, and structure of fructose dehydrogenase

**DOI:** 10.1007/s00216-018-0991-0

**Published:** 2018-03-22

**Authors:** Paolo Bollella, Yuya Hibino, Kenji Kano, Lo Gorton, Riccarda Antiochia

**Affiliations:** 1 Department of Chemistry and Drug Technologies, Sapienza University of Rome, Piazzale Aldo Moro 5, 00185 Rome, Italy; 2 Department of Analytical Chemistry/Biochemistry, Lund University, P.O. Box 124, 221 00 Lund, Sweden; 3 Division of Applied Life Sciences, Graduate School of Agriculture, Kyoto University, Sakyo, Kyoto, 606-8502 Japan

**Keywords:** Fructose dehydrogenase (FDH), Calcium chloride, Direct electron transfer (DET), Enzyme activity, Enzyme structure

## Abstract

We report on the influence of pH and monovalent/divalent cations on the catalytic current response, internal electron transfer (IET), and structure of fructose dehydrogenase (FDH) by using amperometry, spectrophotometry, and circular dichroism (CD). Amperometric measurements were performed on graphite electrodes, onto which FDH was adsorbed and the effect on the response current to fructose was investigated when varying the pH and the concentrations of divalent/monovalent cations in the contacting buffer. In the presence of 10 mM CaCl_2_, a current increase of up to ≈ 240% was observed, probably due to an intra-complexation reaction between Ca^2+^ and the aspartate/glutamate residues found at the interface between the dehydrogenase domain and the cytochrome domain of FDH. Contrary to CaCl_2_, addition of MgCl_2_ did not show any particular influence, whereas addition of monovalent cations (Na^+^ or K^+^) led to a slight linear increase in the maximum response current. To complement the amperometric investigations, spectrophotometric assays were carried out under homogeneous conditions in the presence of a 1-electron non-proton-acceptor, cytochrome *c,* or a 2-electron-proton acceptor, 2,6-dichloroindophenol (DCIP), respectively. In the case of cytochrome *c*, it was possible to observe a remarkable increase in the absorbance up to 200% when 10 mM CaCl_2_ was added. However, by further increasing the concentration of CaCl_2_ up to 50 mM and 100 mM, a decrease in the absorbance with a slight inhibition effect was observed for the highest CaCl_2_ concentration. Addition of MgCl_2_ or of the monovalent cations shows, surprisingly, no effect on the electron transfer to the electron acceptor. Contrary to the case of cytochrome *c*, with DCIP none of the cations tested seem to affect the rate of catalysis. In order to correlate the results obtained by amperometric and spectrophotometric measurements, CD experiments have been performed showing a great structural change of FDH when increasing the concentration CaCl_2_ up to 50 mM, at which the enzyme molecules start to agglomerate, hindering the substrate access to the active site probably due to a chelation reaction occurring at the enzyme surface with the glutamate/aspartate residues.

**Electronic supplementary material:**

The online version of this article (10.1007/s00216-018-0991-0) contains supplementary material, which is available to authorized users.

**Graphical Abstract:**
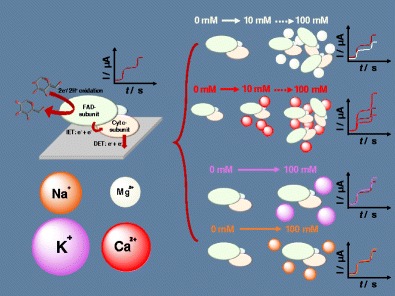
Fructose dehydrogenase (FDH) consists of three subunits, but only two are involved in the electron transfer process: (I) 2e^−^/2H^+^ fructose oxidation, (II) internal electron transfer (IET), (III) direct electron transfer (DET) through 2 heme *c*; FDH activity either in solution or when immobilized onto an electrode surface is enhanced about 2.5-fold by adding 10 mM CaCl_2_ to the buffer solution, whereas MgCl_2_ had an “inhibition” effect. Moreover, the additions of KCl or NaCl led to a slight current increase

## Introduction

In the last 30 years, studies of direct electron transfer (DET) reactions between redox enzymes and electrodes have attracted much interest to understand the background reaction mechanism [1, 2] and thus gain sufficient basic knowledge to be able to establish the basis and to improve the performance of third generation biosensors and enzymatic fuel cells (EFCs) [3–6]. DET-based reactions have been explored for a number of redox proteins [7] such as cytochrome *c* (cyt *c*) [8–11], ferredoxin [12, 13], azurin [14, 15], and redox enzymes [16] such as peroxidases [17–23], hydrogenases [24], “blue” multi-copper oxidases (BMCO) [25–28], sulfite oxidase (SOx) [29, 30], alcohol PQQ dehydrogenase (ADH) [31, 32], cellobiose dehydrogenase (CDH) [33–37], D-fructose dehydrogenase (FDH) [38–41], etc. Several of the redox enzymes mentioned above, such as SOx, ADH, CDH, and FDH are composed of at least two different domains, where one domain is the catalytically active domain containing a bound cofactor such as FAD, PQQ, or MOCO, and a second domain, a cytochrome containing heme *c* or heme *b*, acting as an electron transfer domain or in other words “as a built in mediator” [31] connecting the redox enzyme to its natural electron acceptor, or when immobilized onto the surface of an electrode to the electrode if the enzyme is properly orientated on its surface*.* The electron transfer (ET) reaction between the catalytic and the cytochrome domain, the internal electron transfer (IET) reaction, can be very much dependent on pH, ion strength, and buffer constituents [42]. Studies to enable understanding what determines and limits the ET reactions are fundamental to increase the knowledge about protein structure, mechanisms of redox transformations of protein molecules, and metabolic processes involving redox transformations [43]. DET is the key issue to develop “reagentless” electrochemical biosensors [1–7, 44]. Among the flavocytochrome oxidoreductases, the DET mechanism for membrane bound FDH has not yet been elucidated [45] and therefore FDH has attracted a growing interest also regarding all factors that can influence the DET reaction (e.g., pH, cations, ionic strength, etc.) [46–48].

D-Fructose dehydrogenase (FDH; EC 1.1.99.11) from *Gluconobacter japonicus* NCBR 3260 is a heterotrimeric membrane-bound enzyme complex with a molecular mass of 146.4 kDa, consisting of three subunits/domains [45]: subunit I, which is the catalytic dehydrogenase domain (DH_FDH_) with a covalently bound flavin adenine dinucleotide (FAD) cofactor, where D-(–)-fructose is involved in a 2H^+^/2e^-^ oxidation reaction to form 5-dehydro-D-(–)-fructose; subunit II, which is equivalent to the cytochrome domain (CYT_FDH_) acts as electron acceptor to subunit I and contains three *heme c* moieties covalently bound to the enzyme scaffold and two of them involved in the one-by-one ET pathway [45]; subunit III, which is not involved in the ET but rather plays a key role for the stability of the enzyme complex [38, 45, 49, 50]. FDH exhibits a strict substrate specificity to D-(–)-fructose and therefore has been used as bioelectrocatalyst for biosensor development both in direct and mediated ET (DET and MET) modes [51–54].

Unfortunately, the crystal structure of FDH is not yet available because of the difficulties involved with crystallization of a membrane bound protein with a high molecular weight (ca. 146 kDa). Despite these obvious difficulties, in the last few years much effort has been directed towards considering new crystallization methods [55]. The crystal structure would be of fundamental importance to clarify the ET mechanism of this enzyme with particular attention on the co-factor involved.

**Scheme 1:**
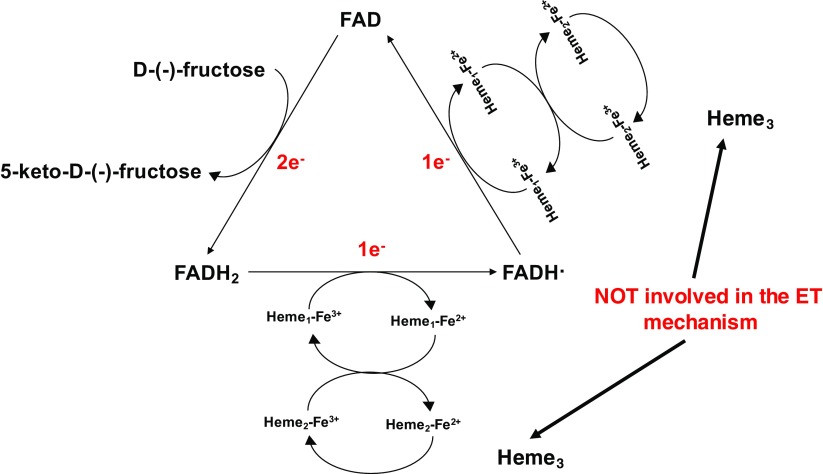
Suggested ET mechanism of the oxidation reaction of FDH. D-(–)-fructose is oxidized to 5-keto-D-(–)-fructose releasing two electrons, which are transferred one by one initially through the FAD, followed by two heme *c* working as monoelectronic acceptors

The suggested ET pathway for FDH when immobilised on the electrode surface and in the absence of any competing e^-^ acceptors [38], is assumed to occur according to Scheme 1:

1. Oxidation of D-(–)-fructose to 5-keto-D-(–)-fructose involving 2e^-^/2H^+^ with the reduction of FAD to FADH_2_;
2. FADH_2_ is sequentially re-oxidized in two separate 1 ET steps. In the first FADH_2_ is partially re-oxidized to FADH**⋅** through the IET pathway between the DH_FDH_ and CYT_FDH_ domains, whereby one of the three heme *c* (heme *c*_1_) is reduced. Next, the electron is transferred from heme *c*_1_ to a second heme *c* (heme *c*_2_) of the two hemes involved in the ET pathway and then to a final electron acceptor, which is the electrode when FDH is adsorbed onto the electrode surface;
3. FADH**⋅** is finally re-oxidized to FAD by heme *c*_1_ and the electron is then transferred to heme *c*_2_ (which gives the second internal electron transfer (IET) step), which in turn is re-oxidized by the electrode whereby FDH is returned to its fully oxidized state.

The influence of ionic strength as well as various cationic and anionic species on enzymes has already been proven to modulate enzymatic activity or stability in different ways [56, 57]; e.g., CDH has two redox domains connected through a flexible linker [58–60], with an ET mechanism similar to FDH but with only one heme in its cytochrome domain as electron acceptor. Moreover, Sezer et al. showed an increase in terms of catalytic current of SOx by increasing the ionic strength of the buffer solution [61], while Feng et al. reported on the influence of the viscosity on the rate of the IET [62]. Nevertheless, each of these enzymes has its own and different dependency on pH and ionic strength because of the amino acids residues at the interface between the subunits connecting each other through linker regions.

The aim of this paper was to investigate whether there is any influence of pH and the concentration of various divalent/monovalent cations on the rate of the IET and the activity and the structure of FDH. First, FDH was immobilized onto graphite electrodes by a drop-casting procedure and amperometric measurements were carried out when shifting the pH in presence of various concentrations of divalent cations (CaCl_2_ and MgCl_2_) or monovalent cations (KCl and NaCl). Furthermore, the influence of pH on the rate of the IET was investigated in the presence of the CaCl_2_ concentration that yielded the highest current density for fructose. These results were compared with those obtained with the spectrophotometric assays carried out in the presence of 2,6-dichloroindophenol (DCIP, bielectronic acceptor) or of cytochrome *c* (cyt *c*, monoelectronic acceptor). Finally, circular dichroism (CD) measurements and homology modelling/docking studies were performed, confirming the hypothesis formulated on the IET of FDH.

## Material and methods

### Chemicals

D-(–)-fructose, calcium chloride (CaCl_2_), magnesium chloride hexahydrate (MgCl_2_ ⋅ 6 H_2_O), sodium chloride (NaCl), potassium chloride (KCl), cytochrome *c* from bovine heart (≥95% based on MW 12,327 Da, prepared using TCA), 2,6-dichloroindophenol (DCIP), sodium acetate (CH_3_COONa, NaAc), 3-(*N*-morpholino)propanesulfonic acid (MOPS), 2-amino-2-(hydroxymethyl)-1,3-propanediol (TRIS), sodium dihydrogen phosphate (NaH_2_PO_4_), hydrochloric acid (HCl), sodium hydroxide (NaOH) were purchased from Sigma Aldrich (St. Louis, MO, USA). D-fructose dehydrogenase from *Gluconobacter japonicus* (FDH; EC 1.1.99.11) was purified from the culture supernatant of *Gluconobacter japonicus* NBRC 3260 obtained from the National Institute of Technology and Evaluation (Nishinomiya, Hyogo Prefecture, Japan) and solubilized in PBS buffer at pH 6 (50~500 mM) containing 0.1 mM 2-mercaptoethanol and 0.1% v/v Triton X-100 (volumetric activity measured with potassium ferricyanide at pH 4.5 = 420 ± 30 U mL^−1^, specific activity = 250 ± 30 U mg^-1^, protein concentration = 1.7 ± 0.2 mg mL^−1^) [49]. All solutions were prepared using Milli-Q water (R = 18.2 MΩ cm at 25 °C; TOC < 10 μg L^−1^, Millipore, Molsheim, France).

### Electrochemical measurements

Graphite rods (Alfa Aesar GmbH and Co. KG, AGKSP grade, ultra “F” purity, and 3.05 mm diameter, Karlsruhe, Germany) were polished on wet emery paper (Turfbak Durite, P1200) and then carefully rinsed with Milli-Q water [56]. The enzyme-modified electrodes were prepared by allowing 5 μL of a FDH solution (volumetric activity measured with potassium ferricyanide at pH 4.5 and found to be 420 ± 30 U mL^−1^, specific activity = 250 ± 30 U mg^-1^, protein concentration = 1.7 ± 0.2 mg mL^−1^) to physically adsorb on the top of the graphite rod electrodes, overnight at 4 °C. The FDH modified graphite electrode, an Ag|AgCl (0.1 M KCl) reference electrode and a platinum wire counter electrode were fitted into a flow-through wall-jet electrochemical cell [63] connected to a flow injection analysis (FIA) system consisting of a peristaltic pump (Gilson, Villier-le-Bel, France) and a six-port valve electrical injector equipped with a 50 μL loop (injection volume 50 μL) (Rheodyne, Cotati, CA, USA) [63]. A potentiostat (Zäta Elektronik, Höör, Sweden) controlled the flow-through cell, and the response of the injected samples was shown on a strip chart recorder (Kipp and Zonen, Utrecht, The Netherlands) [64].

### Spectrophotometric measurements

Two spectrophotometric assays were performed to measure the activity of FDH in solution as a function of both pH and the concentration of cations/ionic strength. In the first assay, the activity of the DH_FDH_ domain was monitored measuring the time-dependent variation of the absorbance at λ = 520 nm (ε = 6.9 mM^-1^ cm^-1^) of a mixture containing 100 μL of 300 mM D-(–)-fructose, 780 μL of 50 mM NaAc buffer, 20 μL of enzyme solution, and 100 μL of 3 mM DCIP as two-electrons/two protons acceptor [56]. In the second assay, the activity of FDH and the internal electron transfer (IET) between the DH_FDH_ and CYT_FDH_ domains was monitored by using the one-electron acceptor cyt *c*, which communicates only with the CYT_FDH_ domain due to steric hindrance because of its dimensions. The absorption is followed at 550 nm (ε = 19.6 mM^−1^ cm^−1^) of a mixture of 20 μL of a 1 mM cyt *c* solution, 100 μL of 300 mM D-(–)-fructose, 860 μL of 50 mM NaAc buffer pH 4.5, and 20 μL of the enzyme solution [65]. One unit of DCIP and cyt *c* activity was defined as the amount of FDH that reduces 1 μmol of DCIP or cyt *c*, respectively, per min under the applied conditions. All measured enzyme activities are the average of three measurements at 25 °C, whereas the average activity values in 50 mM NaAc buffer without additions of “extra” KCl, NaCl, MgCl_2_, or CaCl_2_ were set to 100% and the average activity values in the presence of divalent and monovalent cations in 50 mM NaAc buffer were related to those values. All measurements were carried out using a UV-Vis spectrophotometer 1800 (Shimadzu Europe GmbH, Duisburg, Germany).

### Circular dichroism (CD) measurements

Changes in the secondary structure of FDH as a function of pH and addition of divalent cations to the solution were investigated through CD measurements using a CD spectrometer (J-815 Circular Dichroic Spectrometer, JASCO, Easton, MD, USA) [66]. These measurements were carried out in a 0.1 cm cuvette at 25 °C using a final protein concentration of 0.15 mg mL^-1^, diluted in 50 mM NaH_2_PO_4_ at pH 4.5 (unfortunately we could not use NaAc because the –COOH groups adsorb the light so we needed to use a non-adsorbing medium to obtain reliable data [67]). Then, the concentration of CaCl_2_ or MgCl_2_ was increased in the range of 0–100 mM to receive information about any changes in the secondary structure. The obtained spectra were further fitted with a method called β-structure selection (BeStSel) that takes into account the twist of β-structures for estimation of the secondary structure. This method can reliably distinguish parallel and anti-parallel β-sheets and accurately estimate the secondary structure for a broad range of proteins [68, 69]. Moreover, the secondary structure components applied by the method are characteristic to the protein fold, which in turn can be predicted to the level of topology in the CATH classification from a single CD spectrum.

### Dynamic light scattering (DLS) measurements

The aggregation of FDH in presence of NaCl, KCl, CaCl_2_, MgCl_2_ at different concentrations, 0, 10, 50, and 100 mM was evaluated using Dynamic Light Scattering (Zetasizer Nano ZS90, Malvern Instruments Ltd, Malvern, UK) by considering polydispersion index (PDI) and diameter (nm).

### Homology modeling and docking

The homology models of the individual DH_FDH_ and CYT_FDH_ domains of FDH (GenBank accession number A0A164AQ58_9SYNE) were generated using the Protein Homology/analogY Recognition Engine V 2.0 server (http://www.sbg.bio.ic.ac.uk/) [70] with the individually crystallized DH domains of FAD-glucose dehydrogenase (GDH) from *Aspergillus flavus* (PDB ID 4YNT [71]) and thiosulfate dehydrogenase (TSDBA) from *Marichromatium purpuratum* “as2 isolated” (PDB ID 5LO9 [72]) as templates. The FIREDOCK webserver (http://bioinfo3d.cs.tau.ac.il/FireDock/firedock.html) [73, 74] was used for protein–protein docking of the individual FDH domains. In total, 10 models from three clusters per enzyme were evaluated. The best model has been selected based on the lowest free energy [75]. Structures were visualized using the PyMOL Molecular Graphics System, ver. 1.4 (Schrödinger, New York, NY, USA).

## Results and discussion

### Influence of pH and divalent/monovalent cations on the direct electron transfer (DET) reaction of FDH: electrochemical study

**Fig. 1:**
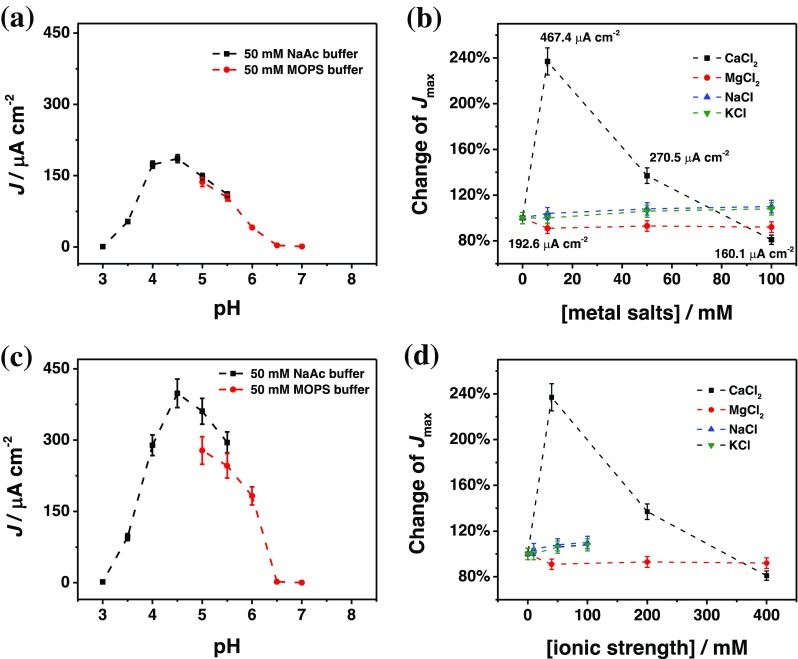
Dependence of the current density on pH **(a)** in the absence and **(c)** in the presence of 10 mM CaCl_2_ carried out in 50 mM NaAc buffer (pH 3–5.5, black), 50 mM MOPS buffer (pH 5–7, red), and 50 mM TRIS buffer (pH 7–10, blue) in the presence of 5 mM D-(–)-fructose; the relative maximal catalytic current densities (*J*_max_) on the concentration **(b)** and ionic strength **(d)** of CaCl_2_ (black), MgCl_2_ (red), NaCl (blue), and KCl (green) solutions (50 mM NaAc buffer pH 4.5) calculated from calibration curves performed in presence of different concentrations of D-(–)-fructose. Experimental conditions: applied potential (E_app_): +0.2 V versus Ag|AgCl_sat_, flow rate: 0.5 mL min^-1^; injection volume 50 μL

In order to study the influence of pH on the DET reaction of FDH with graphite electrodes, we performed amperometric measurements with graphite electrodes simply modified with adsorbed FDH. The measurements were performed with 5 mM D-(–)-fructose as substrate and by varying the pH in the running buffer between 3 and 7 using NaAc buffer between pH 3 and 5.5, MOPS buffer between pH 5 and 7. Figure 1a shows the current density (*J*, μA cm^-2^) dependence on pH. The optimum pH was found at 4.5. The response current rapidly decreased when increasing the pH above 4.5, thus assessing no DET activity starting at pH 7. These results are in good agreement with previous values reported in the literature [76].

Next, the influence of divalent (CaCl_2_ and MgCl_2_) and monovalent (NaCl and KCl) cations was studied through registering calibration curves for D-(–)-fructose at pH 4.5 and varying the salt concentrations in the range of 0–100 mM. Figure 1b shows the dependence of maximum current, *J*_max_, on the concentration of CaCl_2_, MgCl_2_, NaCl, and KCl for FDH at pH 4.5. The *J*_max_ values were obtained from the calibration curves and were set to 100% in the absence of extra added cations. All values obtained in the presence of the different cations are related to those values. Surprisingly, when increasing the concentration of CaCl_2_, a marked increase in *J*_max_ was observed, with a maximum increase of 237%, corresponding to an average maximal current density of 467.4 ± 38.2 μA cm^-2^ for 10 mM CaCl_2_. However, at higher concentrations of CaCl_2_ than 10 mM, an unexpected I_max_ decrease was registered, yielding *J*_max_ values of 140% and of 80%, at 50 mM 100 mM CaCl_2_, respectively. This particular trend for CaCl_2_ is probably due to the presence of side-chain carboxyl functions of surface-exposed aspartic acid (pK_a_ = 3.86) and glutamic acid (pK_a_ = 4.07) residues at the interface of the DH_FDH_ and CYT_FDH_ domains. These –COOH groups are ~90% deprotonated at pH 4.5, creating a strong electrostatic repulsion between the two domains [77]. It can be supposed that a possible complexation of Ca^2+^ with the carboxylic groups of the aspartic and glutamic acid residues takes place, resulting in a closer interaction and shortening the distance between the DH_FDH_ and CYT_FDH_ domains, thus increasing the rate of the IET [58]. On the other hand, the decrease in response signal when the concentration of Ca^2+^ is higher than 10 mM can probably be ascribed to a possible saturation of the aspartate/glutamate residues available at the interface between the DH_FDH_ and CYT_FDH_ domains [78]. Therefore, at concentrations higher than 10 mM, Ca^2+^ is now available for complexation of other aspartate/glutamate residues present on the surface of both subunits. Both the DH_FDH_ and CYT_FDH_ subunits show a partial positive charge at pH 4.5, which might now create a slight repulsion between the two subunits, which is, however, counter-balanced by the attractive interactions. A possible explanation for the inhibition effect observed at 100 mM CaCl_2_ could be the interaction with other enzyme molecules through a Ca^2+^-bridge [79].

Contrary to the effect by addition of CaCl_2_, addition of MgCl_2_ showed a 10% decrease in *J*_max_ already at 10 mM MgCl_2_. This result could be ascribed to a lower affinity of Mg^2+^ compared with Ca^2+^ towards the carboxylic functions available at the interface of the DH_FDH_ and CYT_FDH_ domains. Mg^2+^ is expected mainly to be involved in the complexation reaction occurring between different enzyme molecules (Mg^2+^-bridge), which will partially hinder the substrate access to the active site of FDH and will therefore show a slight inhibition behavior evident already at low MgCl_2_ concentrations. Figure 1c shows that the enhanced effect on the catalytic current density by addition of CaCl_2_ occurs only in the pH range between 3.5 and 5.5 without any shift in the optimum pH of the enzyme activity, due to its presumed effect on the IET as described and discussed above.

In a series of previous investigations, the effect of ionic strength and addition of Ca^2+^ and Mg^2+^ on the catalytic performance of various CDHs was investigated both in solution and when adsorbed on graphite [56, 58, 80, 81]. The results obtained here for FDH are both similar and dissimilar with respect to those obtained for the various CDHs; e.g., for two ascomycete CDHs the addition of both Ca^2+^ and Mg^2+^ had a similar increasing effect on the catalytic efficiency and could even lead to a drastic shift in the pH optimum of the enzyme activity [80]. Thus, it seems as though further investigations on the background reasons for the effect by both ionic strength and by individual cations on the interactions between the various domains participating in the ET pathways in such multi-domain redox enzymes are necessary.

For the monovalent cations, further additions of both KCl and NaCl showed a slight linear increase in *J*_max_ up to 100 mM, which is more evident in Fig. 1d, where the *J*_max_ is plotted versus the ionic strength of the buffer. The concentration of the monovalent cations is directly related to the ionic strength, leading to a general increase in the catalytic current, as already reported in literature for many enzymes, whereas monovalent cations are usually not involved in any complexation reactions occurring at the protein surface [56].

### Influence of pH and divalent/monovalent cations on the catalytic reaction of FDH: spectrophometric study

**Fig. 2:**
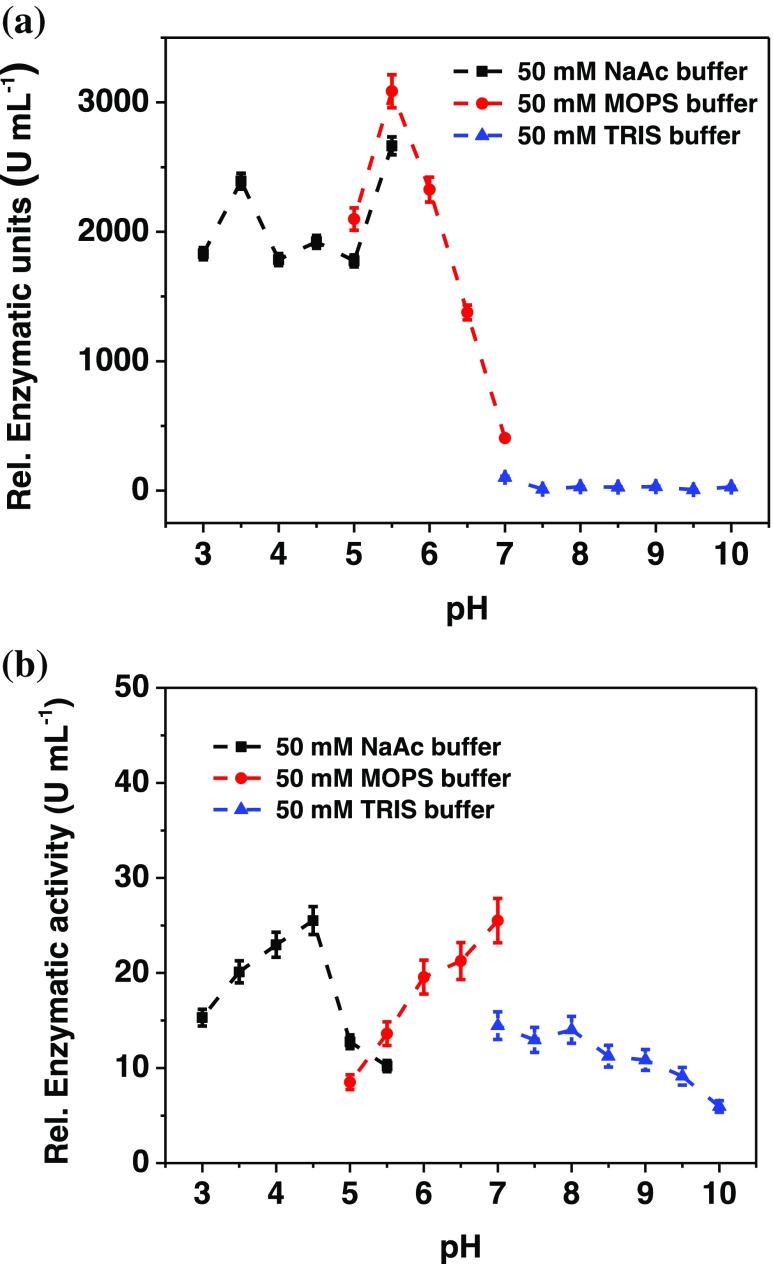
Dependence of the relative activities of **(a)** DCIP and **(b)** cyt *c* on pH carried out in 50 mM NaAc buffer (pH 3–5.5, black), 50 mM MOPS buffer (pH 5–7, red), and 50 mM TRIS buffer (pH 7–10, blue). Experimental conditions: [fructose]: 30 mM, [cyt *c*]: 100 μM, and [2,6-dichloroindophenol, DCIP]: 300 μM

Two different spectrophotometric assays were performed to follow the catalytic reaction of FDH in solution; one with cyt *c* and one with DCIP, acting as mono- and bielectronic acceptors, respectively. DCIP may have a direct connection with subunit I (DH_FDH_), where the catalytic oxidation of D-fructose to 5-keto-D-fructose occurs [59], whereas cyt *c* may interact with both subunits, namely subunits I and II. Figure 2a shows the dependence of the activity of FDH with pH using DCIP as electron acceptor, and one can see that the highest activity is found between 3 and 6 corresponding to an activity of 3086 U mL^-1^ at pH 5.5. For pH values higher than 6, the activity rapidly decreases, indicating a progressive inactivation of DH_FDH_ (subunit I). The dependence of the activity of FDH with pH when using cyt *c* is shown in Fig. 2b. In this case the activity exhibits two pH optima: one at 4.5 and one at 7, in agreement with previous results already reported in the literature [82]. These results could be ascribed to the different mechanisms of the interaction between FDH and cyt *c*: (1) ET may either proceed from the active site (FAD containing subunit I, DH_FDH_) via the heme groups (subunit II containing the three heme centers, CYT_FDH_) toward cyt *c* or (2) directly from the DH_FDH_ to cyt *c*. A similar conclusion was reached when investigating the ET between FDH and cyt *c* by cyclic voltammetry at different scan rates [83, 84].

**Fig. 3:**
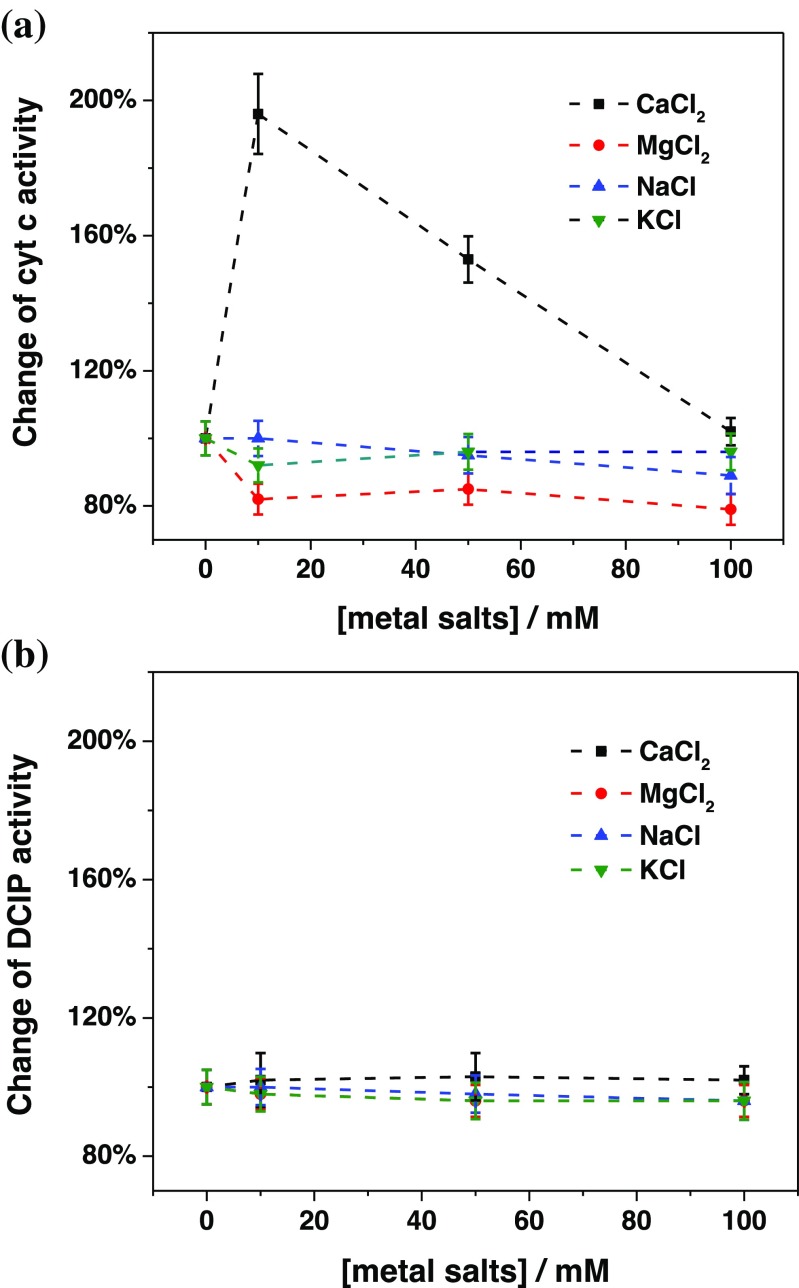
Dependence of the relative **(a)** cyt *c* and **(b)** DCIP activities on different concentrations of CaCl_2_ (black), MgCl_2_ (red), NaCl (blue), and KCl (green) in 50 mM NaAc buffer pH 4.5. Experimental conditions: [fructose]: 30 mM, [cyt *c*]: 100 μM, and [2,6-dichloroindophenol, DCIP]: 300 μM

As far as for the influence of mono- and divalent cations, the results of the spectrophotometric activity assays are shown in Fig. 3a and b. The results obtained with cyt *c* (Fig. 3a) are comparable with those obtained with the amperometric measurements (Fig. 1b) for immobilized FDH on graphite electrodes, as Ca^2+^ ions may affect the rate of the IET and therefore the rate of overall DET reaction. Figure 3b shows that there is no influence of either divalent or monovalent cations on the activity of FDH using DCIP as electron acceptor because DCIP is reduced directly by the FAD deeply buried within the DH_FDH_ (subunit I) and is thus not affected by any change in the IET.

Since the conversion rate of fructose at the FAD is not influenced much by the presence of different ions, the increase in current and cyt reduction found here may have arisen from an enhanced IET and/or a better interaction of the subunit II (heme subunit) with cyt *c* or the electrode.

The obtained results unequivocally confirm the influence of CaCl_2_ on the rate of the IET reaction, whereas there is no influence observed on the catalytic oxidation reaction of D-(–)-fructose at the DH_FDH_ domain by CaCl_2_ or by the other cations tested.

### Influence of CaCl_2_ on the secondary enzyme structure

**Fig. 4:**
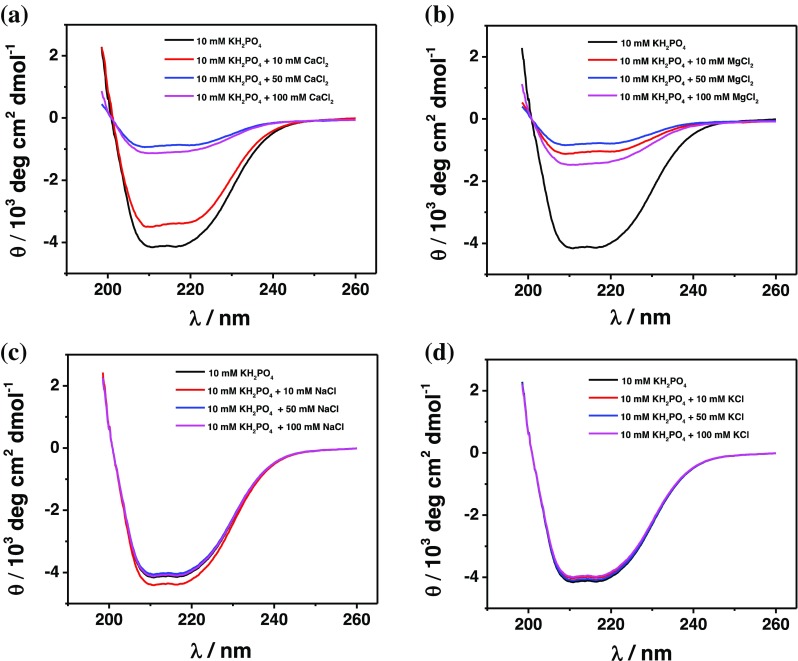
Circular dichroism (CD) spectra of FDH obtained in 10 mM KH_2_PO_4_ pH 4.5 at different concentrations of **(a)** CaCl_2_, **(b)** MgCl_2_, **(c)** NaCl, **(d)** KCl: 0 mM (black), 10 mM (red), 50 mM (purple), and 100 mM (blue)

In order to confirm the results obtained with the amperometric and spectrophotometric assays, CD measurements of FDH were performed increasing the concentration CaCl_2_ or MgCl_2_ in the range of 0–100 mM. Figure 4a–d show the CD spectra recorded for FDH in the presence of CaCl_2_, MgCl_2_, NaCl, and KCl. It is possible to observe that the secondary structure only to some extent shows minor changes up to a concentration of 10 mM, whereas at higher concentrations of CaCl_2_ the spectra reveal large changes that can be explained by agglomeration with other FDH molecules due to Ca^2+^-bridge interactions between enzyme molecules, as shown in Fig. 4a. Therefore, at low CaCl_2_ concentrations, it is possible to observe an intra-complexation caused by Ca^2+^ ions, which might increase the rate of the IET reaction, whereas at high CaCl_2_ concentrations inter-complexation reactions occur due to aggregation of enzyme molecules caused by the high CaCl_2_ concentration. Conversely, the CD spectra in the presence of low Mg^2+^ concentration already showed large changes, probably due to inter-complexation reactions occurring between different enzyme molecules, as depicted in Fig. 4b. Moreover, Na^+^ and K^+^ did not show any influence on the secondary enzyme structure, as reported in Fig. 4c and d, because they are not involved in any chelation reactions normally occurring with divalent cations [85]. Finally, formation of FDH multimers or aggregates was clearly proven by using dynamic light scattering (DLS) measurements as reported in Figs. S1–S4 and Table S1 in the Electronic Supplementary Material (ESM).

### Structural analysis and docking of FDH domains

**Fig. 5:**
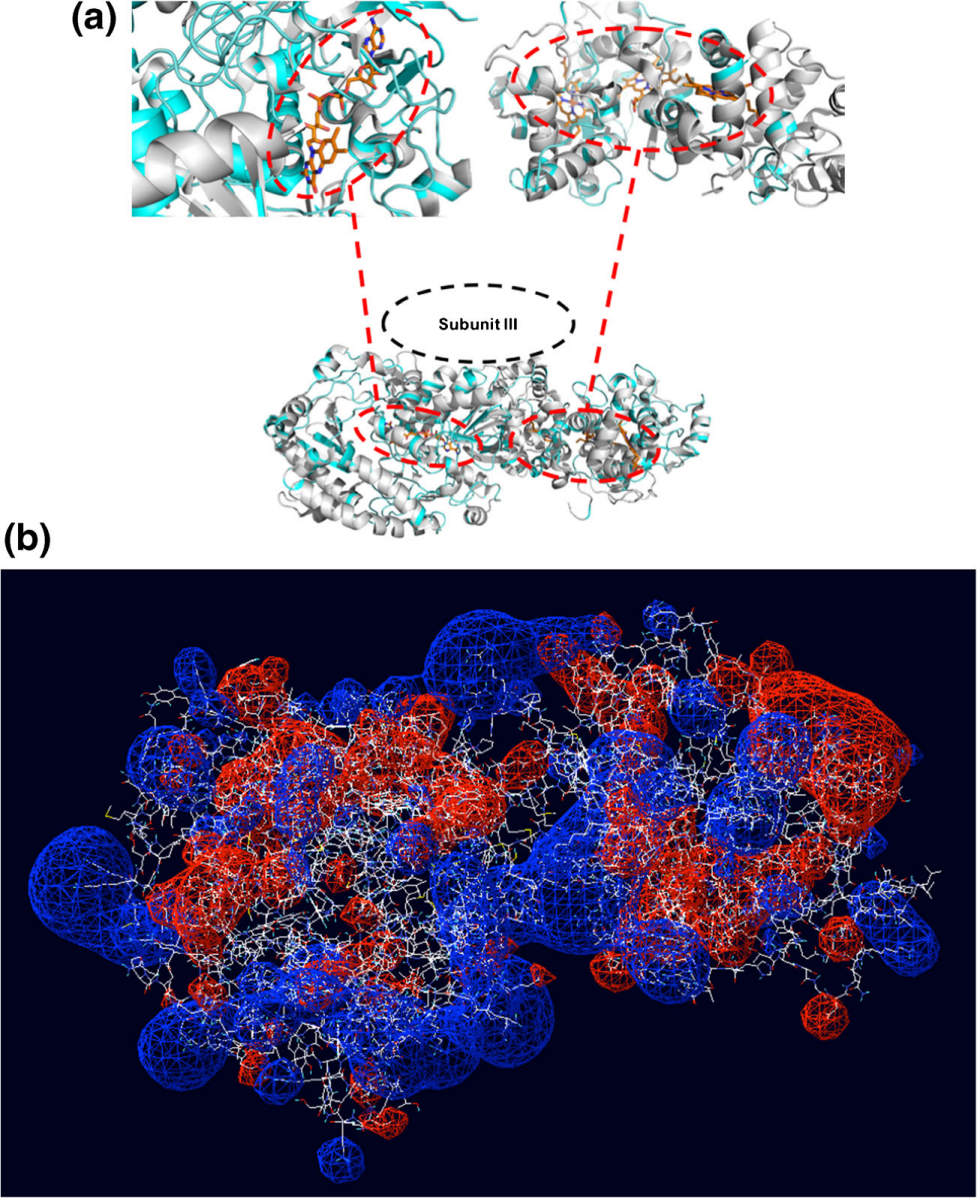
**(a)** Detailed representation of structural alignment between Subunit I of FDH (homology model, grey structure) and *Aspergillus niger* FAD-glucose dehydrogenase (PDB ID: 4ynt, light blue structure); and between Subunit II of FDH (homology model) and thiosulfate dehydrogenase (tsdba) from *Marichromatium purpuratum* “as2 isolated” form (PDB ID: 5lo9); **(b)** electrostatic map of Subunits I and II after docking obtained from the homology models

The already characterized FAD-glucose dehydrogenase from *Aspergillus flavus* (4YNT) and thiosulfate dehydrogenase (TSDBA) from *Marichromatium purpuratum* “as2 isolated” (5LO9) were used for modeling and docking studies of the DH_FDH_ and CYT_FDH_ domains, respectively. Figure 5a shows the homology models of the DH_FDH_ and CYT_FDH_ domains highlighting in the insets the position of the FAD cofactor (subunit I) and the three heme *c* (subunit II), respectively, while subunit III was not possible to model because of the absence of a similar structure. From previous studies [49] it was already stated that subunit III has no influence on the DET reaction but it is important for the overall stability of the enzymatic complex.

Moreover, the docking between the DH_FDH_ and CYT_FDH_ domains was needed to highlight those amino acids expected to be involved in the interaction between the two domains, viz., Asp, Glu, Lys, Arg, and Tyr. DH_FDH_ exhibits five amino acid residues negatively charged at the interface, whereas CYT_FDH_ contains three such amino acid residues. The occurrence of these residues could partially explain the reason for the highest rate of the IET reaction at pH 4.5, where the repulsion between the two domains is lowest, whereas at pH between 7 and 10 the repulsion between the two domains is high and will cause the low rate of the IET reaction. However, this model does not give any information about Ca^2+^-bridging interactions. Nevertheless, a concentration of 10 mM CaCl_2_ results in the upper limit in terms of an enhanced IET, probably because only a few possible matches between the negatively charged amino acid residues and Ca^2+^ ions are found at the interface between the DH_FDH_ and the CYT_FDH_, while the number of negatively charged amino acids residues exposed on the other parts of the enzyme surface is much higher, confirming the possibility of other Ca^2+^-bridging interactions between different individual enzyme molecules allowing the aggregation process [86, 87].

## Conclusions

This paper demonstrates the possibility to enhance the measurable activity of FDH either in solution or when immobilized onto an electrode surface about 2.5-fold by adding 10 mM CaCl_2_ to the buffer solution, whereas MgCl_2_ had no such effect. Additions of KCl or NaCl led to a slight linear increase in *J*_max_ of about 10%. However, CaCl_2_ had an effect only at pH 4.5 because there was no shift in the optimum pH, in contrast to what was shown for ascomycete CDHs in previous papers [56, 58, 80, 81]. Moreover, the amperometric and spectrophotometric results were confirmed through conformational changes observed in the secondary structure of FDH, revealing unfolding of the protein occurring at high CaCl_2_ concentrations (50 and 100 mM) followed by aggregation.

By the homology models, it was assumed that Ca^2+^ at lower concentrations was chelated by the few amino acid residues negatively charged, shortening the distance between the DH_FDH_ and CYT_FDH_ domains, leading to an enhanced IET, while at higher concentrations of Ca^2+^, they give Ca^2+^-bridging interactions with other enzyme molecules.

These results show that studies of the mechanism of the IET between the DH_FDH_ and CYT_FDH_ domains are of great interest to shed further light into the physiological functions of FDH, as well as for the development of third generation biosensors and enzymatic fuel cells based on FDH.

## Electronic supplementary material

ESM 1 (PDF 133 kb)
