# Supplementary material for: The influence of pH and divalent/monovalent cations on the internal electron transfer (IET), enzymatic activity, and structure of fructose dehydrogenase
Source: Anal Bioanal Chem. 2018 Mar 22;410(14):3253–64. doi: 10.1007/s00216-018-0991-0 (PMC5937911; doi:10.1007/s00216-018-0991-0)
Supplement: Supplementary file 1 — (PDF 133 kb) [file 216_2018_991_MOESM1_ESM.pdf]

Analytical and Bioanalytical Chemistry

Electronic Supplementary Material

The influence of pH and divalent/monovalent cations on the internal electron transfer (IET), enzymatic activity and structure of fructose dehydrogenase

Paolo Bollella, Yuya Hibino, Kenji Kano, Lo Gorton, Riccarda Antiochia

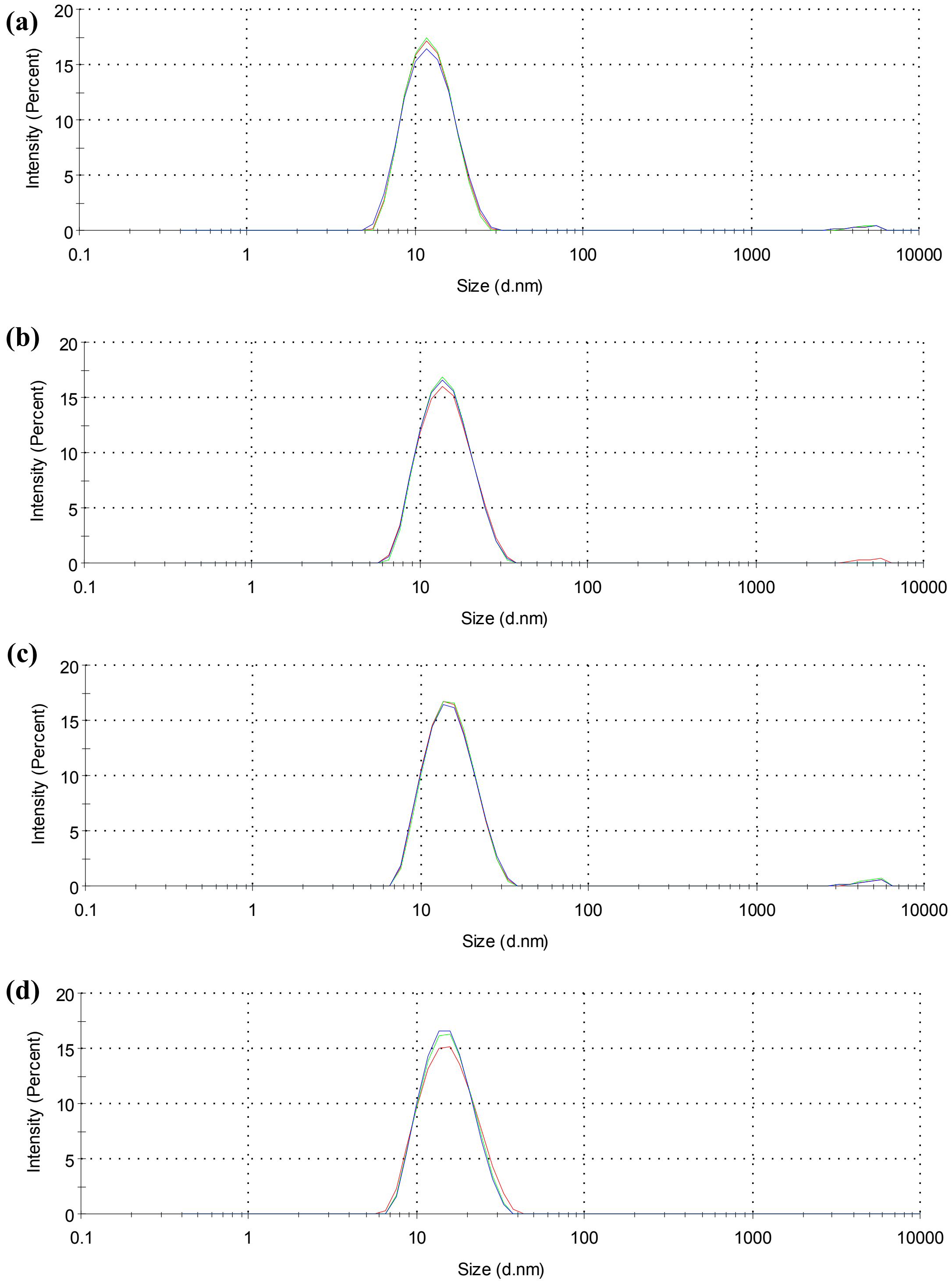

Fig. S1 DLS measurements performed with a 0.15 mg mL^{-1} solution of fructose dehydrogenase (FDH) from *Gluconobacter japonicus* in 10 mM NaH_2PO_4 pH 4.5 (3 repetitions, 12 runs) at different NaCl concentrations: 0 mM (a), 10 mM (b), 50 mM (c) and 100 mM (d)

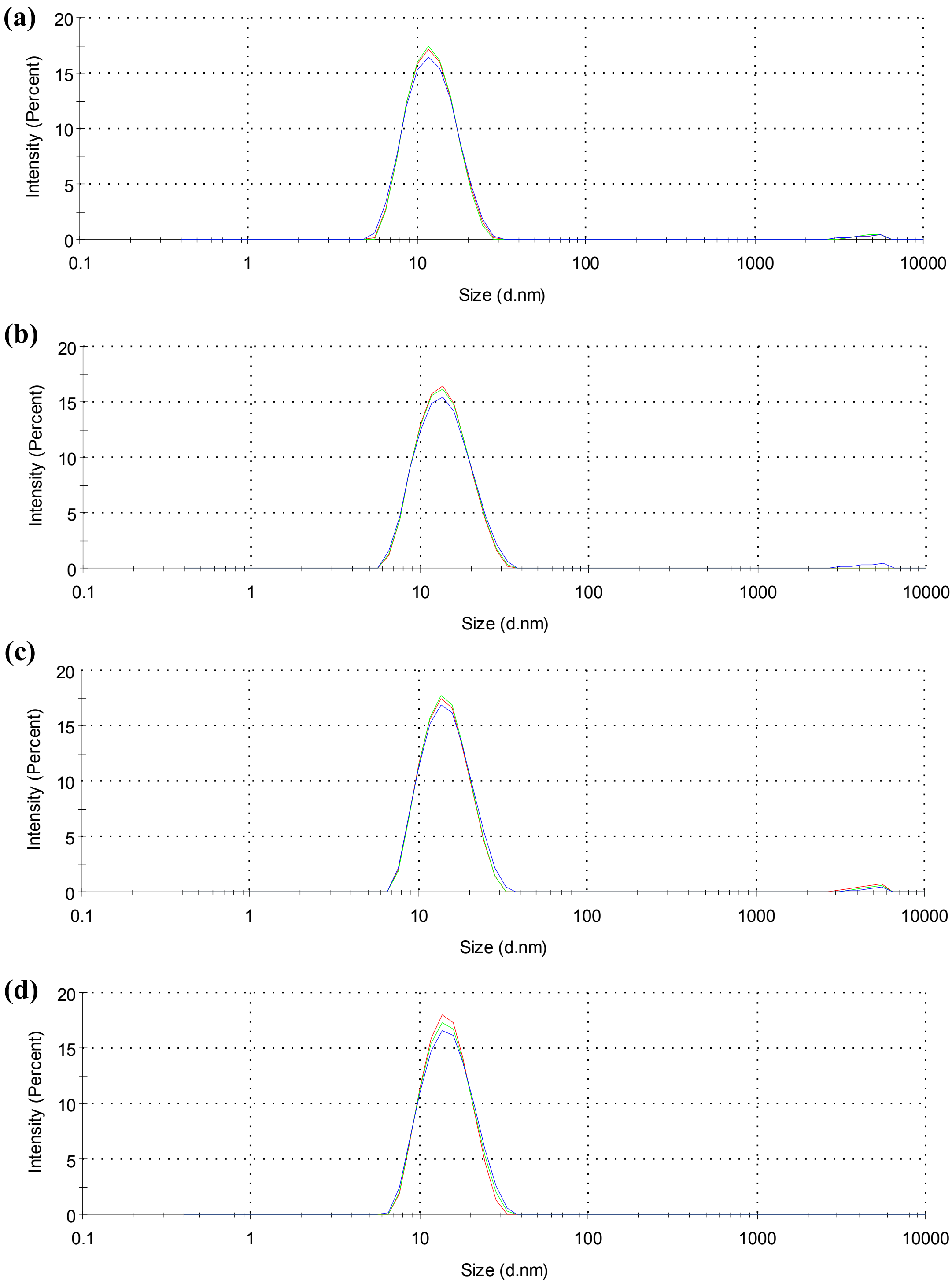

Fig. S2 DLS measurements performed with a 0.15 mg mL^{-1} solution of fructose dehydrogenase (FDH) from *Gluconobacter japonicus* in 10 mM NaH_2PO_4 pH 4.5 (3 repetitions, 12 runs) at different KCl concentrations: 0 mM (a), 10 mM (b), 50 mM (c) and 100 mM (d)

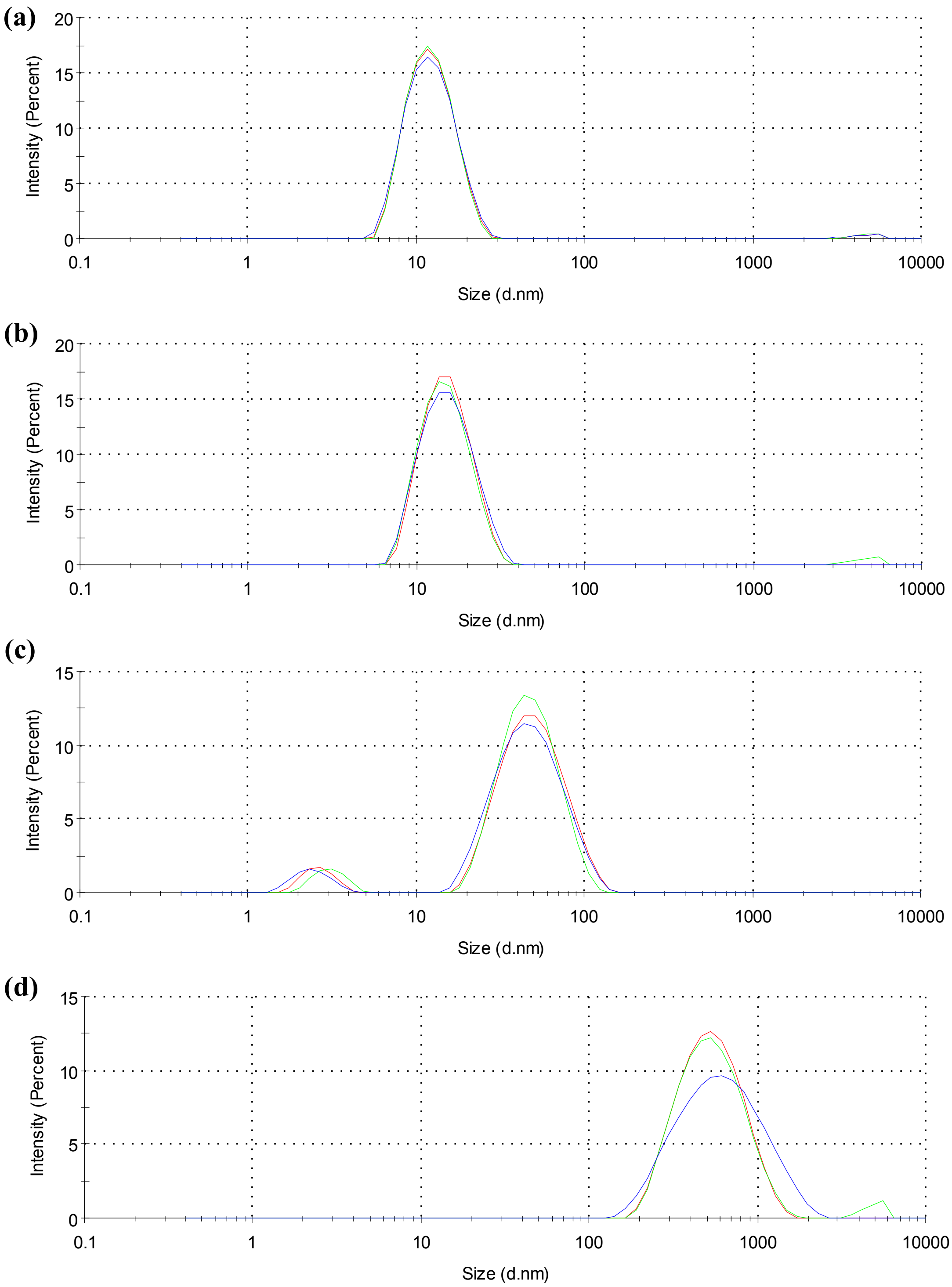

Fig. S3 DLS measurements performed with a 0.15 mg mL^{-1} solution of fructose dehydrogenase (FDH) from *Gluconobacter japonicus* in 10 mM NaH_2PO_4 pH 4.5 (3 repetitions, 12 runs) at different $CaCl_2$ concentrations: 0 mM (a), 10 mM (b), 50 mM (c) and 100 mM (d)

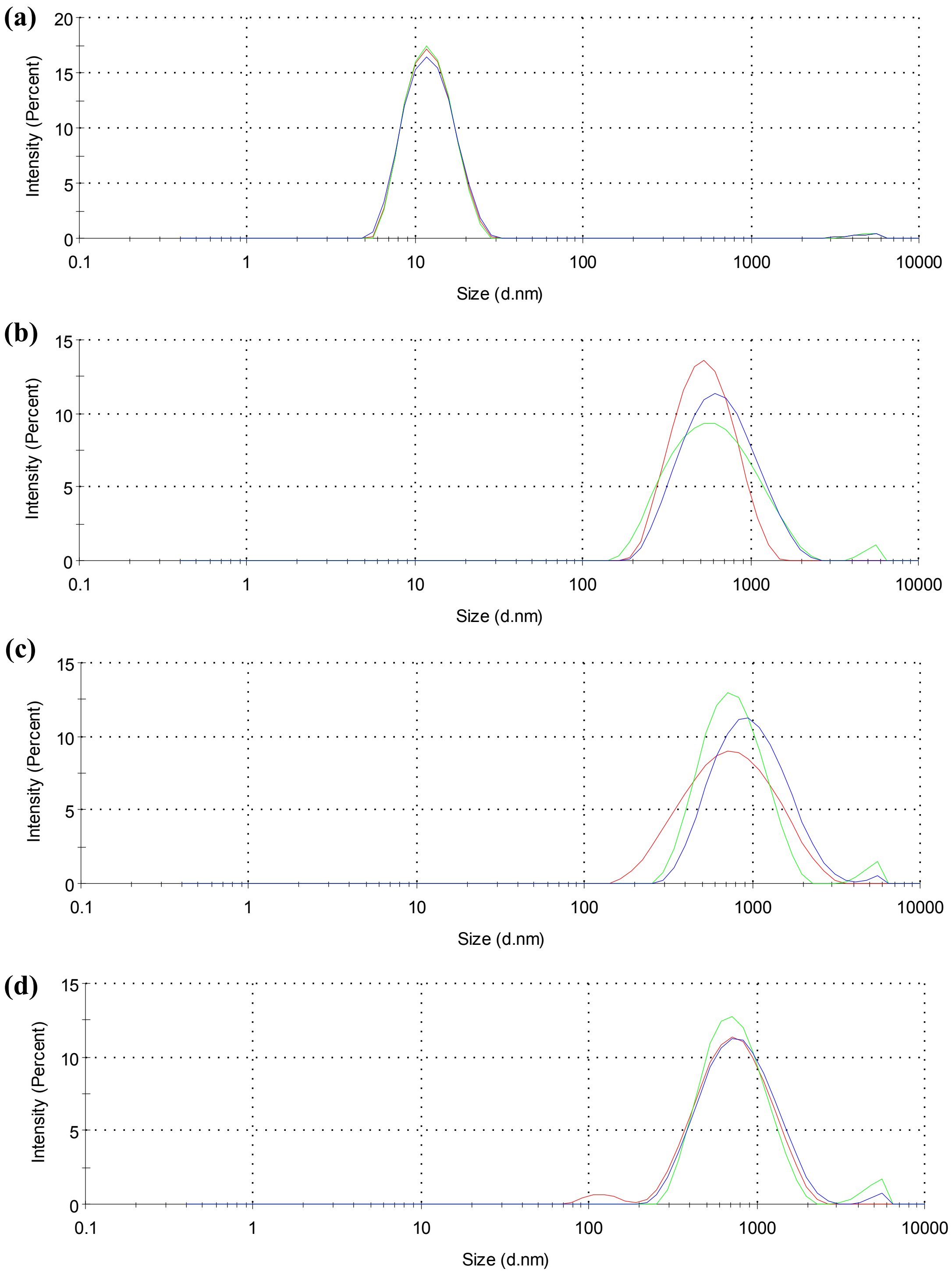

Fig. S4 DLS measurements performed with a 0.15 mg mL^{-1} solution of fructose dehydrogenase (FDH) from *Gluconobacter japonicus* in 10 mM NaH_2PO_4 pH 4.5 (3 repetitions, 12 runs) at different $MgCl_2$ concentrations: 0 mM (a), 10 mM (b), 50 mM (c) and 100 mM (d)

Table S1 DLS measurements performed with a 0.15 mg mL^{-1} solution of fructose dehydrogenase (FDH) from *Gluconobacter japonicus* in 10 mM NaH_2PO_4 pH 4.5 (3 repetitions, 12 runs) at different NaCl, KCl, $CaCl_2$, $MgCl_2$ concentrations

**Buffer 10 mM NaH_2PO_4 pH 4.5**	**PDI**	**d.(nm)**
**No salt added**	0.145 ± 0.005	12.6 ± 0.2
**10 mM NaCl**	0.135 ± 0.003	14.8 ± 0.1
**50 mM NaCl**	0.154 ± 0.005	15.5 ± 0.1
**100 mM NaCl**	0.123 ± 0.008	15.9 ± 0.2
**10 mM KCl**	0.134 ± 0.011	14.3 ± 0.1
**50 mM KCl**	0.148 ± 0.014	14.9 ± 0.2
**100 mM KCl**	0.091 ± 0.011	15.1 ± 0.2
**10 mM $CaCl_2$**	0.130 ± 0.004	15.6 ± 0.3
**50 mM $CaCl_2$**	0.311 ± 0.007	52.4 ± 1.5
**100 mM $CaCl_2$**	0.244 ± 0.012	606.7 ± 67.0
**10 mM $MgCl_2$**	0.240 ± 0.045	652.5 ± 79.2
**50 mM $MgCl_2$**	0.292 ± 0.027	915.1 ± 85.1
**100 mM $MgCl_2$**	0.273 ± 0.009	811.5 ± 35.9
